# Cancer‐associated mutations in endometriosis reframe a benign disease through molecular oncology

**DOI:** 10.1002/1878-0261.70295

**Published:** 2026-09-23

**Authors:** Clarissa Mujacic, Maria Concetta Vitale, Rita Siino, Emilia Di Giovanni, Carla Ferrante Bannera, Enrico Di Marco, Andrea Gottardo, Tancredi Didier Bazan Russo, Nadia Barraco, Alessandro Perez, Valerio Gristina, Antonio Galvano, Giuseppe Badalamenti, Antonio Russo, Viviana Bazan, Lorena Incorvaia

**Affiliations:** ^1^ Department of Precision Medicine in Surgical, Oncological, and Critical Care (Me.Pre.C.C.), Section of Medical Oncology University of Palermo Italy; ^2^ Department of Biomedicine, Neuroscience and Advanced Diagnostics (BIND), Section of Medical Oncology University of Palermo Italy

**Keywords:** cancer‐associated mutations, endometriosis, gynaecological cancer, molecular pathology, oncogenic pathways, precision medicine

## Abstract

Endometriosis is a benign yet debilitating condition, and its molecular complexity remains unexplored. Although clinically recognized, its genetic landscape has long been overlooked. This review analyses cancer‐associated mutations (CAMs) identified in endometriotic lesions, revealing parallels with malignancy. Recurrent mutations in key oncogenic and tumour suppressor genes—*PIK3CA*, *CTNNB1*, *KRAS*, *PTEN* and *ARID1A*—emerge with patterns distinct from cancer, yet suggesting shared mechanisms. Despite the limited genomic data, these findings position endometriosis as a valuable model for investigating early tumour‐like processes. By reframing endometriosis through the lens of molecular oncology, this study offers new insights into its pathogenesis and proposes a translational framework for advancing diagnostics and understanding disease progression at the benign–malignant interface.

## Graphical abstract

This review aims to comprehensively analyse cancer‐associated somatic mutations (CAMs) present in endometriotic lesions, emphasizing their biological roles, spatial distribution and implications for translational applications in medicine. By contextualizing a benign state within a genomic framework, this analysis seeks to establish its value as a unique model for investigating precancerous characteristics and the advancement towards malignancy.

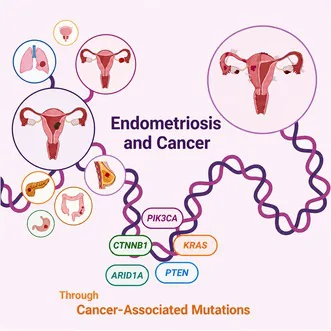

**Abbreviations**
*ARID1A*
: AT‐Rich Interactive Domain‐containing protein 1A
ATR: Ataxia Telangiectasia and Rad3‐related protein
CAMs: Cancer‐Associated Mutations
COSMIC: Catalogue Of Somatic Mutation In Cancer
*CTNNB1*
: Beta‐Catenin 1
*HDAC6*
: Histone‐deacetylase‐6
KLF6: Krüppel‐like Factor 6
*KRAS*
: Kirsten RAt Sarcoma virus
PARP: Poli ADP‐ribose polymerase
*PIK3CA*
: Phosphatidyl‐Inositol‐4,5‐bisphosphate 3‐Kinase Catalytic subunitα
*PTEN*
: Phosphatase and TENsin homolog deleted on chromosome 10
PVs/LPVs: Pathogenic/Likely‐Pathogenic Variants
SWI/SNF: SWItch/Sucrose Non‐Fermentation
WHO: World Health Organization

## 1 Endometriosis: The etiology and its cancer‐like features

The endometrium constitues the mucous membrane of the uterus. The tissue is divided into a basal layer, where the uterine glands and capillarization of the arteries are located, and a functional layer, containing the ciliated epithelial lining and the most superficial layers of the lamina propria, characterized by connective tissue poor in fibres and rich in vessels and glands. The endometrial functional layer, under the influence of ovarian hormones, exhibits significant cyclic variability, representing a tissue of considerable dynamism [1]. Endometriosis is a pathological condition impacting this tissue. Up to March 2023, the World Health Organization (WHO) counted 6–10% of the female population as endometriosis‐affected, with data revealing an increasing trend. The disease is a chronic inflammatory condition characterized by the presence of epithelial and stromal cells, histologically similar to those characterizing the endometrium, but located in different tissues and organs [https://www.who.int/].

**Fig. 1:**
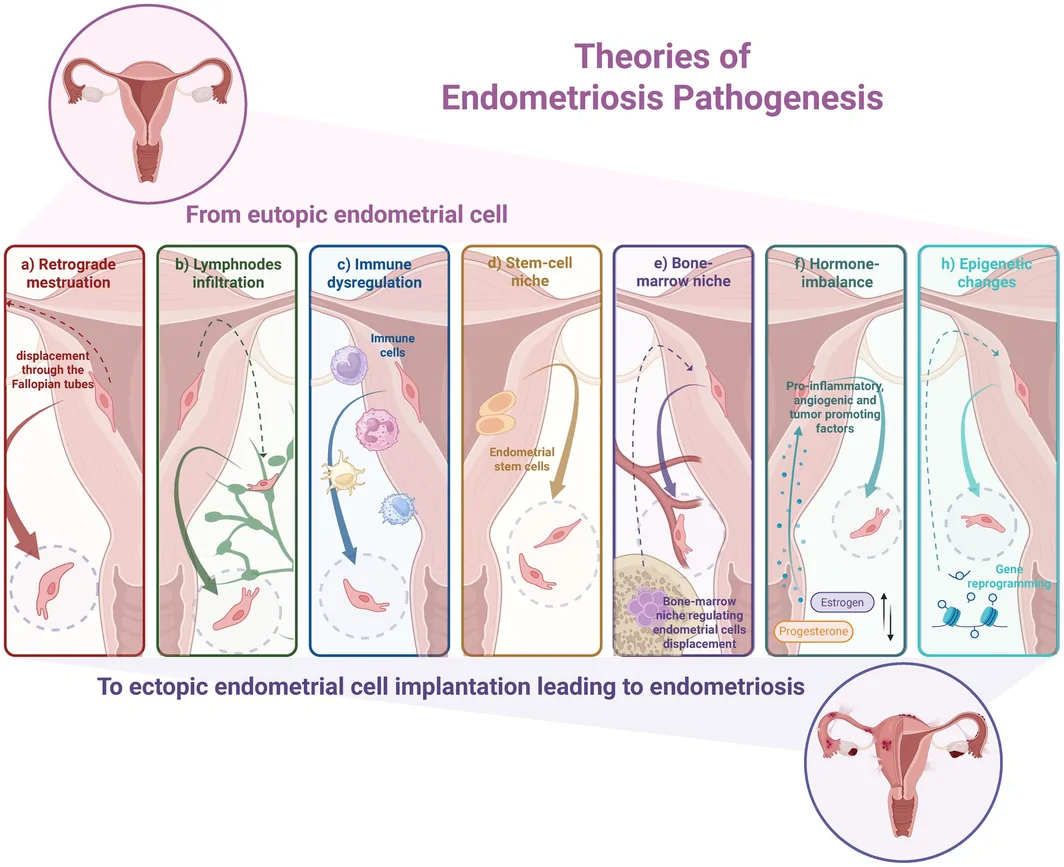
Theories of endometriosis pathogenesis. Graphic illustration of the seven main theories uncovered over the last decades. (A) The retrograde menstruation theory, which principally explains ovarian and superficial peritoneal endometriosis, sees endometrial cells of the menstrual fluid ‘walking’ through the fallopian tubes, establishing over the lesion supported by angiogenetic processes. (B) The benign metastasis theory supports endometriotic infiltration of lymph nodes and distant endometriotic lesions (e.g. lungs) with endometrial cell dissemination and proliferation through the lymphatic system. (C) The immune dysregulation mechanism is based on proinflammatory pathways that block apoptosis, leading to endometrial cell proliferation and outgrowth. (D) Endometrial stem cell theory—based on the stem cells present in the inner layer of the original tissue associated with endometrial regeneration of the normal period—suggests that the latter are capable of stimulating cell growth in ectopic tissues, leading to the establishment of endometriosis. (E) The variant theory of bone marrow cells, which can physiologically regenerate endometrial tissue, seems to be able to misplace cells in ectopic regions through CXCL12/CXCR4 axis dysregulation. (F) Hormonal dysregulation theory, either associated with oestrogen upregulation and sensitization or progesterone resistance, leads to non‐responsive endometrioid lesions growth, stimulated by pro‐inflammatory cytokines and angiogenic/tumour‐promoting factors. (G) Hormones dysregulation that, associated with other molecular epigenetic changes, from DNA methylation to histone acetylation or different RNA transcription, enables genetic reprogramming and endometriosis development, bringing up the last interesting theory [4].

Although its origin is still unclear, various theories have been developed from the first most diffused ‘retrograde menstruation’ theory to other major ones [2] (Fig. 1).

Lesions are classified into stages, although these do not correlate with the severity of symptoms [3, 4].

The endometrium is also the site of endometrial cancer development, which is one of the most common gynaecological cancers. A real breakthrough in such neoplasms occurred in 2013, when a new molecular classification was introduced in clinics, changing the knowledge and treatment landscape [5, 6].

Inspired by the fact that both diseases originate from the same endometrial cells, the role of molecular biology and genetics in oncology and the changes these have brought to clinical practice with precision therapies, we evaluated how a similar approach could be applied to a disease with interesting characteristics that warrant further investigation.

Genetic oncology is based on the search for somatic mutations in genes known to be predictive and prognostic biomarkers of disease. The characteristics investigated and summarized in this review are for the field of endometriosis.

Such alterations could reveal variations in endometriosis, potentially changing patient care and disease handling.

## 2 Cancer‐associated mutations or CAMs

At a molecular level, there are the so‐called ‘cancer‐associated mutations’ (CAMs) genes, which occur in known cancer‐linked genes that play a role in neoplasm development. Examples are *PTEN*, *KRAS*, *POLE*, *CTNNB1* as well as *TP53* or *PIK3CA* [6]. Our starting point is this biological context.

CAMs involved in malignant processes, such as overgrowth, tissue invasion and immune escape, are mutated in cases of endometriosis. However, endometriosis remains a benign condition that stands at the intersection of physiology and pathology, causing chronic damage. It does not progress to malignancy, but its molecular behaviour mirrors that of cancer in many ways. This restrained transformation offers a unique perspective: by studying what endometriosis lacks to become malignant, we can better understand what drives cancer towards its aggressive nature.

In this review, we explore the five major cancer‐associated genes—*PIK3CA, CTNNB1, ARID1A, KRAS* and *PTEN*—identified in the literature of endometriosis and registered in the Catalogue Of Somatic Mutation In Cancer (COSMIC) database as the most frequently mutated genes in endometriosis. Their biological significance, the mutations found and any parallels with oncology are reported here, in order to provide an overview of the different molecular information for a disease that is still largely unexplored.

Starting from established knowledge in oncology, this molecular description could pave the way for a deeper understanding of endometriosis and open new perspectives for translational research.

Endometriosis emerges as a model of resistance to fatal transformation, despite sharing key molecular pathways with cancer. This may offer valuable insights into oncological mechanisms, highlighting potential targets for future diagnostic, prognostic and therapeutic strategies.

## 3 CAMs of endometriosis

### 3.1 PIK3CA

The Phosphatidyl‐Inositol‐4,5‐bisphosphate 3‐Kinase Catalytic subunit Alpha (*PIK3CA*) gene codes for the PI3K enzyme, a protein of the *PI3K/Akt/mTOR* pathway, which controls cell growth, proliferation and migration and is mostly mutated in colorectal and breast cancers [7]. This pathway plays a decisive role in the TCGA molecular classification of endometrial cancer [5, 6].

**Fig. 2:**
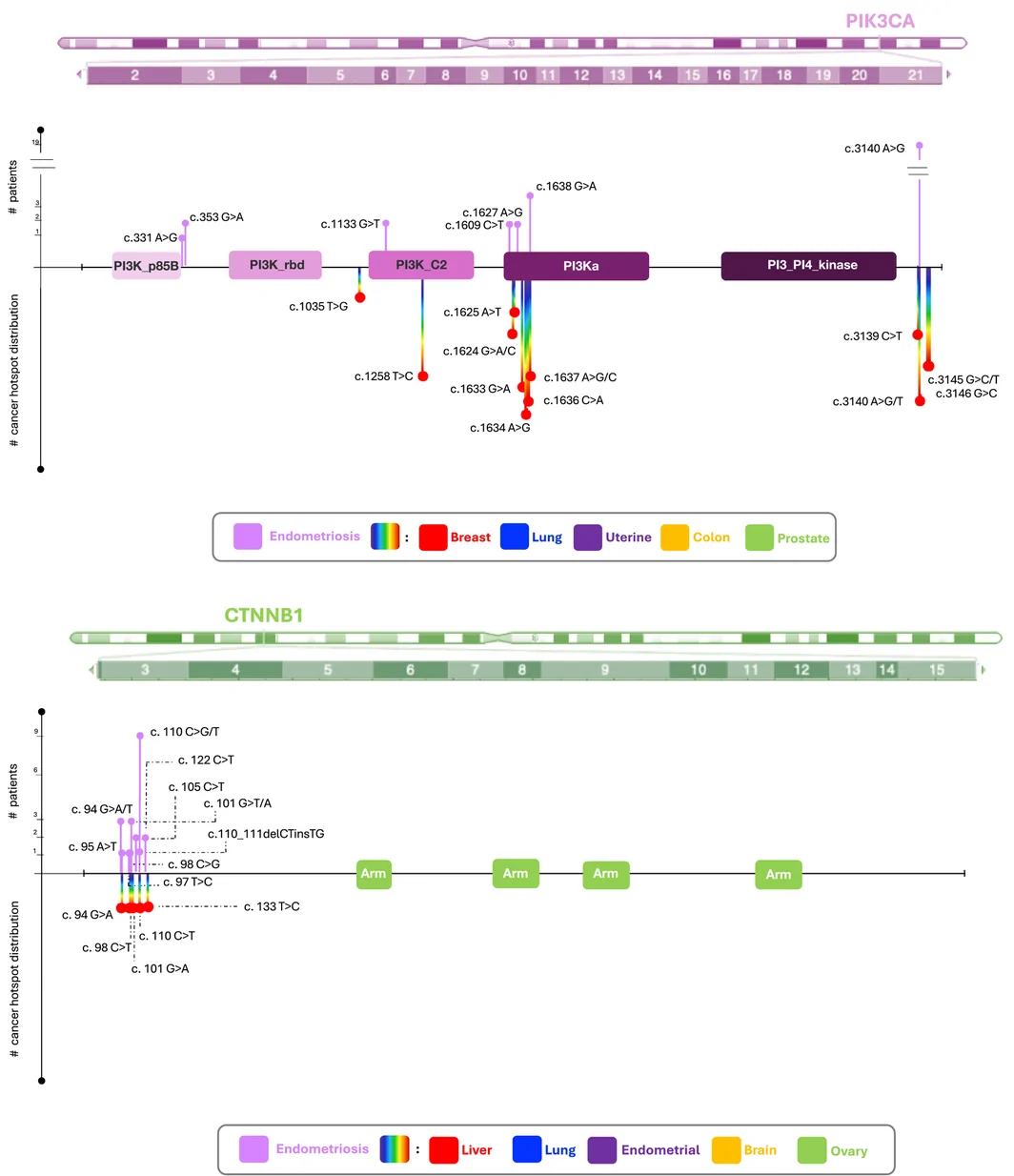
*PIK3CA* and *CTNNB1* point mutation distributions. Distribution of gene point mutations in the COSMIC endometriosis cases database (upper part of the graph) versus the main cancer hotspot point mutations occurring in the same gene described in oncology.

In the COSMIC database, the kinase was mutated in 24% of registered cases (27 over 113) [https://cancer.sanger.ac.uk/cosmic]. All patients present missense or nonsense mutations, already classified as pathogenic/likely pathogenic variants (PVs/LPVs) in multiple tumour types, able to hyperactivate the pathway [https://varsome.com] [Fig. 2].

Despite previous studies in which CAMs were described as very rare and almost absent in endometriosis compared to endometrial or ovarian cancer, recent data suggest in‐line results [8, 9, 10, 11, 12].

In 2017, exon sequencing analysis of tissue samples revealed CAMs in cases of endometriosis without cancer, including a *PIK3CA* mutation. The hotspot mutation c.3127A>G was identified and classified as an LPV [8].

Another study of women with iatrogenic endometriosis identified 32.2% of patients presenting CAMs in their tissues. In this context, one case reported a PV mutation in *PIK3CA*, c.3140 A>G (H1047R) [10].

In 2018, Suda et al. demonstrated the heterogeneity and diversity of the endometrial epithelium. CAMs were found in both endometriotic and uterine endometrial epithelium; *PIK3CA* was found mutated in 23% of endometriotic patients (3 out of 13), while its prevalence in the normal endometrium was different, with a 63% prevalence (7 out of 11 samples). Despite the limited number of cases studied, a significant difference emerged: the physiological epithelium presented a mutation in the kinase well distributed over the whole gene sequence, associated with physiological changes caused by the ageing process, while in endometriosis samples, the mutation occurred in the Adaptor‐Binding Domain (PIK3_p85B—Fig. 2), impairing protein–protein interactions and leading to ectopic site outgrowth [11].

A different interesting comparison of endometrioma cysts revealed that the group with a higher immunostaining score was associated with CAMs overexpression, among which *PIK3CA*, which had an increased significance in the malignant tendency of Ovarian Clear Cell Carcinoma [12].

The gene relevance in oncology and its growing incidence have led to the design of *PIK3CA* inhibitors: from Pan‐*PIK3CA* inhibitors to Isoform‐specific PIK3CAi [13]. Given their efficacy, such inhibitors could represent a valuable tool against severe endometriosis cases if they present cancer‐like molecular features.

### 3.2 CTNNB1

The Beta‐Catenin 1 (*CTNNB1*) gene encodes the beta‐catenin protein, which coordinates cell adhesion and regulates gene transcription [14].

Its role as an intracellular transducer is linked to the highly conserved *Wnt* signalling pathways that determine cell communication. Indeed, during tumorigenesis, the *Wnt* pathway dysregulation leads to less anchor dependency and easier metastasis due to the sustained morphological changes [15].

In the COSMIC database, 29% of cases showed *CTNNB1* mutations, with three patients presenting mutations in both *CTNNB1* and *PIK3CA* and one overlapping with the *ARID1A* gene [https://cancer.sanger.ac.uk/cosmic] [Fig. 2].

All cases present missense mutations; frequently in exon 3 with variants classified as PV/LPVs [https://varsome.com—Fig. 2].

In a study of iatrogenic endometriosis cases, only one patient in a cohort of 76 was found to have a mutation. The mutation presented was c.101G>T, which still occurred in exon 3, leading to upregulation of the protein [10]. However, the iatrogenic origin of the lesions limits the value of this information.

Despite the limited knowledge, the growing incidence of *CTNNB1* mutations in endometriosis deserves mention. Indeed, different *in vitro* preclinical studies have highlighted gene susceptibility to hormones, other proteins or inhibitors.

Beta‐catenin expression leads to endometriotic cell invasiveness and diffusion capacity, both *in vitro* and in xenotransplanted immunodeficient mice experiments [16].

The transcription factor Krüppel‐like Factor 6 (KLF6) was recently discovered to decrease the proliferation, migration and angiogenesis of ectopic endometrial cells through beta‐catenin inhibition [17]. A signalling that was successfully downregulated by two different inhibitors (*ICG‐001* e *C82*): *in vitro* experiments showed decreased endometriotic stromal cell proliferation, fibrogenesis and migration, while promoting apoptosis, and *in vivo* endometriotic lesion growth lowering, proving a role of the pathway in sustaining endometriosis growth [18].

Its role in endometrial cancer, endometriosis and endometriosis‐associated ovarian cancer was also demonstrated in a study where *CTNNB1* mutations were identified in 56% of the ovarian endometrioid carcinoma cases (28 out of 50 patients) and in 41.9% of coexisting endometriosis lesions (26 over 62 cases) [19]. *CTNNB1* mutations only partially overlapped between the two conditions.

A study of seven different cell lines, varying from lung, colon and ovarian carcinomas, and a related xenograft model of the *CTNNB1*‐mutant lung cancer line proved the efficacy of TTK inhibitors against tumour growth. This molecule acts on the spindle assembly checkpoint kinase TTK (Mps1), which is fundamental for chromosome segregation fidelity. Experiments have shown that the *CTNNB1* mutant status increases TKK cellular sensitivity to treatment by five times [20]. Recent studies on hepatocarcinoma and endometrial cancer have supported this inhibitory function, proving the efficacy of *CTNNB1* molecular targeting [21, 22].

### 3.3 ARID1A

The AT‐Rich Interactive Domain‐containing protein 1A (*ARID1A*) is a tumour suppressor gene, a p53 collaborator acting over *CDKN1A* and *SMAD3*, regulating tumour growth and cell proliferation [https://www.ncbi.nlm.nih.gov/clinvar/]. *ARID1A* mutations and loss of expression have been reported in ovarian clear cell carcinomas and uterine endometrioid cancers, especially in uterine low‐grade endometrioid carcinoma [5, 23].

*ARID1A* is one of the main components of the SWItch/Sucrose Non‐Fermentation (SWI/SNF) complex, which remodels DNA accessibility. Its transcriptional regulation capacity takes over different pathways, from DNA repair mechanisms to immune microenvironment influencing and signalling pathways, such as the *PI3K/AKT/mTOR* pathway, which is normally reactivated with the co‐occurrence of *ARID1A* and *PIK3CA* mutations [24]. Gene downregulation leads to a high physiological disequilibrium.

**Fig. 3:**
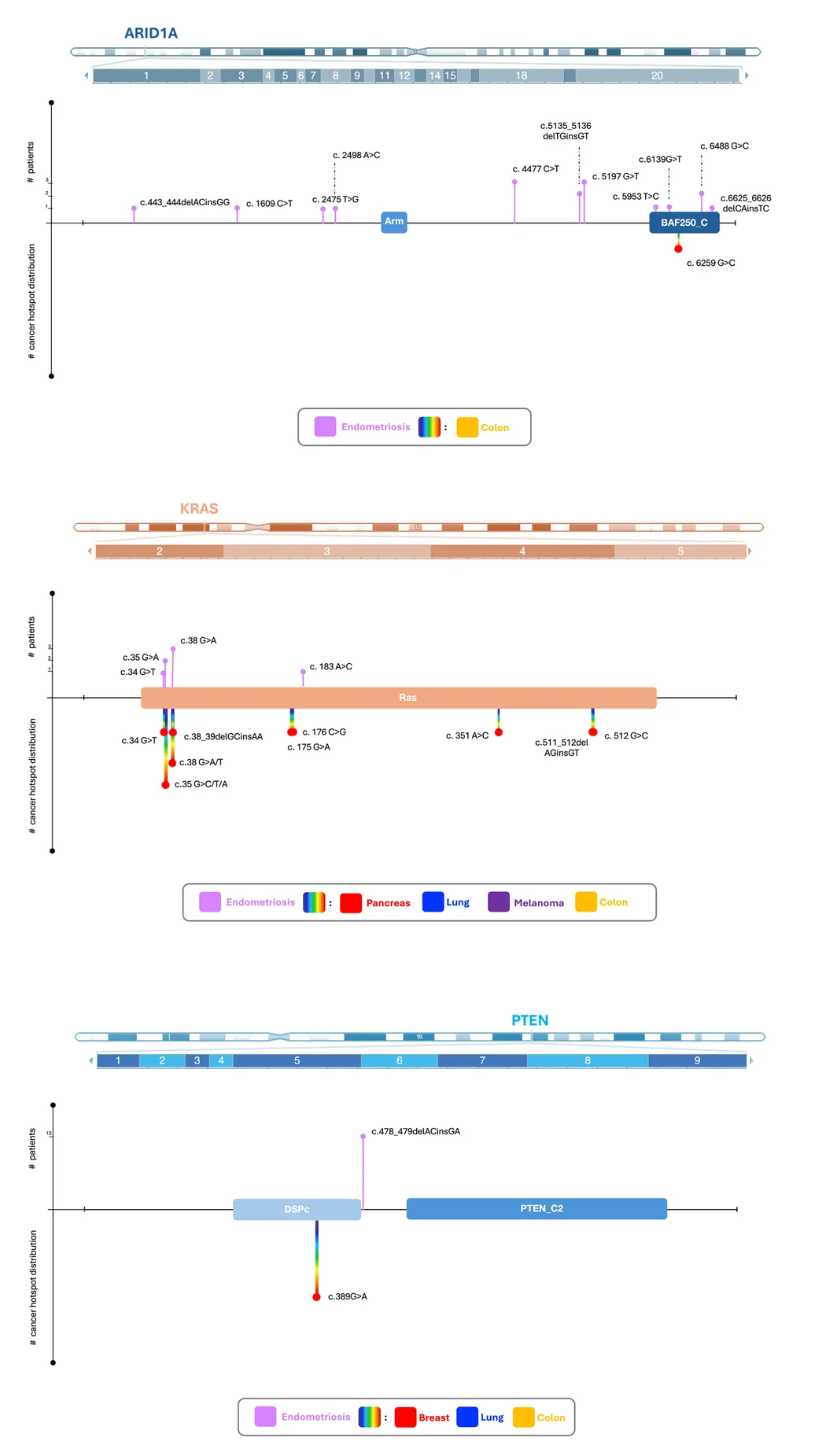
*ARID1A, KRAS* and *PTEN* point mutation distribution. Distribution of gene point mutations in the COSMIC endometriosis cases database (upper part of the graph) versus the main cancer hotspot point mutations occurring in the same gene described in the oncology literature (lower part of the graph).

In the COSMIC matrix, 19% of the samples were *ARID1A* mutated. Among these, nonsense substitution and frameshift deletion were reported [https://cancer.sanger.ac.uk/cosmic] (Fig. 3).

In the endometriosis setting, *ARID1A* mutations are not well characterized.

A study showed how *ARID1A* downregulation is more accentuated under oxidative stress conditions, present in endometriosis cases [25].

Other data proved *ARID1A*'s role in this disease through the *PI3K/AKT* pathway: the occurrence of the gene mutation with consequent pathway alterations has been seen in endometriosis cases as well as in endometriosis‐associated ovarian carcinomas [26]. In this and other cancer settings, *ARID1A*'s involvement in relation to *PIK3CA* has been marked multiple times.

An interesting single‐cell analysis of endometriosis samples suggested that *ARID1A* is a growth promoter of local angiogenic and lymphatic endothelial cells through paracrine signalling modulation [27].

Although most studies have been cancer‐related, different studies have proven its role in reproductive and hormone‐sensitive tissues. As an oncosuppressor, but mostly for its role in the SWI/SNF complex, this gene seems to represent an interesting molecular target.

Although no specific drugs act on it, several components have been hypothesized. Among these, Histone‐deacetylase‐6 (*HDAC6*), PARP (Poli ADP‐ribose polymerase) and ATR (Ataxia Telangiectasia and Rad3‐related protein) inhibitors are of particular interest, as *ARID1A* is involved in the Homologous Recombination pathway [28, 29]. In line with these findings, possible therapies in *ARID1A*‐mutated patients could be offered.

### 3.4 KRAS

Kirsten RAt Sarcoma virus (*KRAS*) is a highly conserved gene in the small GTPase superfamily. Its studies are related to multiple malignancies, from lung adenocarcinomas to colorectal cancers. *KRAS*, along with *NRAS* and *HRAS*, plays an important role in cell proliferation and cell death evasion [30].

In COSMIC, 17% of patients showed *KRAS* mutations. Among those, most cases presented LPVs/PVs mutations all located in the same codons of cancer hotspot regions [https://cancer.sanger.ac.uk/cosmic] (Fig. 3).

A study on cancer‐associated mutations in endometriosis without cancer revealed consistent results. Exon sequence analysis showed one case with co‐occurring mutations in the two different lesions sequenced; the c.35G>T (p. G12V) and the c.35G>C (p. G12A). PCR analysis of the other four mutated patients revealed that they all present LPVs/PVs variants. Interestingly, mutations were always in the endometriotic glandular epithelium and not in the stroma or normal eutopic endometrial epithelium [8]. Such histological differences have been sustained by multiple studies, including at the single‐cell level [11, 27, 28].

The *KRAS* literature related to endometriosis offers numerous insights.

A study focusing on *KRAS* codon 12 variants confirmed the presence of *KRAS* mutated cases, with varying mutated percentages from 27.6% (8/29 patients) in stage I endometriosis to 58.1% (25 of 43) of stage IV cases. While pain severity was not seen to be dependent on the *KRAS* status, surgical difficulties, in relation to higher endometriosis anatomic severity, resulted in KRAS dependence [30].

RNA *in situ* hybridization evaluating p.G12V *KRAS* in ovarian endometriosis showed 38.4% of mutated cases in a cohort of 26 patients. Here, *KRAS*‐mutated patients were associated with higher inflammation levels, consistent with the gene's activity [31].

Some studies showed co‐occurrence of *KRAS* and *PIK3CA* mutations; indeed, *in vitro* models harbouring both oncogene mutations were linked to enhanced cell migration, invasion and proliferation of endometriosis cell lines, leading to more aggressive forms. This response was associated with the inflammatory LOX/PTX3 pathway: their siRNA knockdown reduced the migration activity, suggesting alternative molecular targets for endometriosis [32].

Effective *KRAS* therapies are not operative in the clinic, except for the allele‐specific inhibitor against *KRAS* G12C, which traps the mutated molecule in its inactive form. Other pan‐KRAS therapies are currently being studied and can inactivate *KRAS* mutated cells by blocking the nucleotide exchange of its activation, independent of the mutation, both *in vitro* and *in vivo* models [33].

### 3.5 PTEN

Lastly, the Phosphatase and TENsin homologue deleted on chromosome 10 (*PTEN*) gene mutations. The protein tyrosine phosphatase PTEN is a well‐known oncosuppressor gene that acts on the *PIK/Akt/PKB* pathway, regulating cell growth, survival and proliferation [34]. A total of 9% of samples were registered as *PTEN* mutated [https://cancer.sanger.ac.uk/cosmic] (Fig. 3).

The first insight into *PTEN* involvement in endometriosis dates back to two decades ago, with a study demonstrating how the loss of such an oncosuppressor led to endometrial cancer from benign endometriotic cysts [35]. This result was later debated when *PTEN* and *EMX2* were not statistically associated with endometriosis formation [36].

In contrast, the role of *PTEN* in endometriosis development via apoptosis and angiogenesis regulation has been proven with its overexpression both *in vitro* and *in vivo* [37].

In addition, in progesterone‐resistant women with endometriosis, the miRNA *miR‐92‐a*, negatively correlated with *PTEN* levels, played a role in the disease: the miR‐92‐a antagomir efficiently resumed progesterone treatment response in immunodeficient mice by suppressing stromal cell proliferation and reducing ectopic tissue lesions [38].

Despite the low percentage of endometriosis‐mutated cases, *PTEN* is the most frequently mutated gene in endometrial cancer.

Pharmacologically *PTEN* is difficult to target. Several studies have attempted to act on its loss of function. On the preclinical side, multiple experiments are trying to overcome *PTEN* non‐functionality by acting over its related pathways, among all Akt/mTOR, lowering tumour growth in cases of endometrial and prostate tumours. On the clinical side, multiple trials at different phases are evaluating different drug efficiencies, from multiple ATP competitors to PARP inhibitors. To date, no *PTEN*‐targeting drug has been used in clinics [39].

## About endometriosis and endometrial cancer: The plots

Although the same genes described as altered in oncology were analysed, the patterns were still different. In particular, when compared to data from endometrial cancer which is associated with cells of the same nature, different prevalence and mutations can be seen in the endometriosis setting. Although the comparison of cases between endometrial cancer and endometriosis is disproportionate, with endometriosis representing only 2% of cases compared to cancer, Table 1 includes some benchmarks [https://cancer.sanger.ac.uk/cosmic].

Despite the unbalanced comparison of cases between endometrial cancer and endometriosis—which accounts for one‐fiftieth of the cases recorded compared to cancer —, in Table 1 some benchmarks listed [https://cancer.sanger.ac.uk/cosmic].

**Table 1:** Endometriosis versus endometrial cancer mutations. Gene mutations distribution in the COSMIC endometriosis cases database of over 423 samples (left) compared to gene mutations distribution in the COSMIC endometrial cancer database of 20 569 samples (right)—endometrial cancer mutation rates confirmed with the cBioPortal [https://www.cbioportal.org].

	Endometriosis	Endometrial cancer
*PIK3CA*	24% (27 out of 113)	29% (1348 out of 4699)
*CTNNB1*	29% (21 out of 73)	19% (540 out of 2913)
*ARID1A*	19% (15 out of 78)	31% (559 out of 1779)
*KRAS*	17% (7 out of 42)	16% (703 out of 4460)
*PTEN*	9% (12 out of 129)	43% (1615 out of 3747)

*PTEN* and *ARID1A* are more diffused and altered in cancer cases, with over a third of cases presenting at least one mutation. In contrast, these two CAMs were not the ‘top‐two’ frequently mutated ones in endometriosis. Differently, *CTNNB1* had a minor frequency of mutated cases in neoplasms compared to endometriotic lesions, while *KRAS* presented almost the same percentage. *PIK3CA* was inferior, but not significantly, in endometriosis cases compared to the endometrial cancer cases.

Interestingly, not mentioned on the ‘top 20 gene’ list of endometriosis cases is *TP53*, the oncosuppressor mutated in 28% of endometrial cancer patients [https://cancer.sanger.ac.uk/cosmic].

Despite discussing the same CAMs, the ones that are conspicuously higher mutated in cancer are both oncosuppressor genes, controlling cell survival or death.

Also, the endometriosis mutational matrix shows a maximum of two hits per patient, while the endometrial cancer matrix of mutated genes exposes a complex and higher mutational status: every patient is mutated in at least three genes, the most maintained among all are *PTEN*, *PIK3CA* and *TP53*, highlighting how multiple hits maintain cellular instability and neoplasms development [https://cancer.sanger.ac.uk/cosmic].

## Clinical trials: Insights into the clinic

Research over the US ClinicalTrials.gov library revealed—among interventional studies, from Phase II to completed trials with available results—35 endometriosis clinical studies [https://clinicaltrials.gov]. Since endometriosis is a benign condition, all trials focus on alleviating women's suffering and pain. Most trials evaluated the effects of hormone‐controlling drugs, mostly in ‘moderate to severe’ and painful endometriosis cases (Table 2).

**Table 2:** US clinical trials. Description of the main clinical trials.

Drug	Study population	Study description	Target	Mechanism of action	Trial identifier	Notes
Phase IV						
Ulipristal	Women with chronic, endometriosis‐related pelvic pain	Pilot Study	Progesterone (PR) receptor antagonist	Blockage of progesterone contraceptive effect on the ovary and on the endometrium	NCT02213081	
Resveratrol	Women with endometriosis diagnosed by laparoscopy	Randomized, prospective, double blind with two arms	NQO2 (alone and in interaction with AKT1), GSTP1, oestrogen receptor beta, CBR1, and integrin αVβ binder	Antioxidant activity, via antioxidant enzymes and blockage on DNA damage by free radicals	NCT02475564	
Phase III						
Relugolix	Women with endometriosis participants of the MVT‐601‐3101 (NCT03204318) or MVT‐601‐3102 (NCT03204331) trials	Open‐label, single‐arm, long‐term efficacy and safety extension study	Gonadotropin (Gr) Hormone Releasing antagonist	Blockage of Gr release	NCT03654274	Co‐administration with low‐dose estradiol (E2) and norethindrone acetate (NETA)
Linzagolix	Women with endometriosis moderate to severe endometriosis‐associated pain who have already completed 6 months of Linzagolix treatment (75 mg alone or of 200 mg in combination with ABT)	Prospective, randomized, double‐blind study	GrHR antagonist	Blockage of Gr release	NCT04335591	
Linzagolix	Women with histological diagnosis of pelvic endometriosis up to 10 years before screening and with moderate to severe endometriosis‐associated pain during the screening period	Multicentre, randomized, double‐blind, placebo‐controlled, clinical study	GrHR antagonist	Blockage of Gr release	NCT03992846	
Elagolix	Premenopausal female women with moderate to severe endometriosis‐associated pain		GrHR antagonist	Blockage of Gr release	NCT03213457	Administered in combination with estradiol or norethindrone acetate
Elagolix	Premenopausal women with moderate to severe endometriosis‐associated pain who completed the 6‐month treatment period in the pivotal study M12‐665 (NCT01620528)	Multicentre, double‐blind randomized study	GrHR antagonist	Blockage of Gr release	NCT01760954	
Triptorelin pamoate	Chinese women with endometriosis	Multicentre, randomized, open‐label, parallel, active‐controlled study	Luteinizing hormone‐releasing hormone (LHRH) agonist	Blockage of LH and FSH production	NCT03232281	Compared to estradiol suppression
Phase II						
OBER2109	Women with moderate‐to‐severe endometriosis associated pain	Prospective, dose‐finding, randomized, parallel group, double‐blind, placebo‐controlled study	GrHR antagonist	Blockage of Gr release	NCT02778399	
BGS649	Women with moderate to severe endometriosis	Randomized, double‐blind, double‐dummy, placebo‐controlled study	Aromatase inhibitor	Blockage of oestrogen synthesis	NCT01190475	
Cabergoline	Young women (15–40 years) with surgically confirmed endometriosis	Randomized pilot study	Dopamine D2 receptor agonist	Blockage of prolactin hormone release from the pituitary gland	NCT02542410	With norethindrone acetate as control
Elagolix	Women with chronic, endometriosis‐related pelvic pain and surgical diagnosis	Randomized, double‐blind, placebo‐ and active‐controlled study	GrHR antagonist	Blockage of Gr release	NCT00797225	Compared to placebo and leuprorelin (an approved endometriosis therapy)
Norethindrone acetate + conjugated Oestrogens	Young women (13–22 yrs) with surgically confirmed endometriosis	Randomized trial	Progestin	Blockage of PR receptors	NCT00474851	Added with oestrogens administration
Medroxyprogesterone acetate	Young women (18–49 yrs) with surgically confirmed endometriosis	Randomized, double‐blind, active‐controlled study	PR receptor antagonist	Blockage of the GnRH secretion	NCT00437658	Comparison with placebo and Elagolix
Telapristone acetate	Premenopausal women with pelvic pain associated with endometriosis confirmed within the last 7 years and using prescription analgesics for symptomatic pain	Multicentre, three‐arm, parallel design, randomized, double‐blind study	PR receptor antagonist	Blockage of the GnRH secretion	NCT01728454	
MT‐2990	Moderate to severe endometriosis‐related pain in women with surgically diagnosed endometriosis	Randomized, double‐blind, placebo‐controlled study	Interleukin‐33 antibody	Decreased inflammation, lesion proliferation and fibrosis	NCT03840993	
Gefapixant	Premenopausal female participants with moderate to severe endometriosis‐related pain	Proof of concept, randomized, double‐blind, placebo‐controlled clinical trial	P2X3 antagonist	Blockage of sensory circuits	NCT03654326	
Botulinum toxin	Women (18–50 yrs) with pain associated and pelvic muscle spasm and endometriosis	Randomized trial	Acethilcoline (Acth) antagonist	Blockage of presynaptic release of Acth, inducing paralysis and atrophy	NCT01553201	

An overview of these studies showed the wide use of GnRH antagonists (e.g. Linzagolix, Elagolix) or different targets such as progesterone receptors or luteinizing hormone‐releasing agonists [https://clinicaltrials.gov] (Table 2).

Among the studies screened, the MT‐2990 and the Gefapixant trials presented interesting mechanisms of action that caught our attention.

MT‐2990 acts on IL‐33 targeting endometriosis inflammation (NCT03840993), while Gefapixant, based on a *P2X3* antagonist, is able to target pain sensory circuits without demonstrating any efficacious effect (NCT03654326) [40, 41, 42].

Except for the immunotherapy treatment (MT‐2990), there are no other non‐hormonal completed trials reported [https://clinicaltrials.gov].

On the still ongoing active trials, none are evaluating the effect of drugs already used in clinical oncology practice, based on specific target mutations also reported here and present in endometriosis lesions; more studies are, instead, interestingly focusing on microRNAs, mostly to guarantee better diagnosis [https://clinicaltrials.gov].

The WHO International Clinical Trials Registry Platform did not show interesting related ongoing clinical trials related to the topic [https://www.who.int/clinical-trials-registry-platform].

Differently, the European Union Clinical Trials Register brought up some other new but not still effective treatments (Table 3) [https://www.clinicaltrialsregister.eu].

**Table 3:** European Union Clinical Trials Register. Description of the main clinical trials.

Drug	Study population	Study description	Target	Mechanism of action	Trial identifier (EudraCT number)	Notes
BAY 1128688	Women with symptomatic endometriosis over a 12‐week treatment period	A randomized, placebo‐controlled, double‐blind, parallel‐group, multi‐center, exploratory dose–response study	Alpha‐Keto Reductase family 1 member C3 (AKR1C3)	Prostaglandin levels reduction	BAY1128688/17472 NCT03373422	
PGL5001	Women with peritoneal and/or ovarian endometriosis with an inflammatory component.	Phase IIa study	JNK (c‐Jun N‐terminal kinases)	JNK inhibition	2012‐001219‐22	With concomitant administration of depot medroxyprogesterone acetate (DMPA)
BAY 1817080	Women with symptomatic endometriosis	Phase 2b randomized, double‐blind, open for active comparator, parallel‐group, multicentre study	P2X3	P2X3 antagonist	2020‐003131‐16	
ZK 811752	Women with symptomatic endometriosis	A multicentre, double‐blind, randomized, placebo‐controlled, parallel‐group study	C‐C chemokine receptor type 1 (CCR1)	CCR1 antagonist	2004‐000630‐37	

In addition, none of the drugs cited have been clinically approved, according to the European Society of Human Reproduction and Embryology (ESHRE) guidelines which offer analgesics, surgery or hormone therapy as the only available treatments [43].

## 4 Conclusion

Overall, our analysis confirmed the presence of cancer‐associated mutations (CAMs) in endometriosis. In this review, we provide a molecular overview of endometriosis, emphasizing cancer‐related genes that are altered in the lesions and their potential contribution in the aetiopathogenesis and sustained progression of the disease. Although the functional impact and penetrance of these mutations within a specific context remain to be elucidated, their recurrence highlights intriguing parallels with oncogenic processes. We explored a selection of key genes, outlining their biological implications and potential roles in the cellular behaviour characteristics of this condition, such as proliferation, invasion and immune evasion.

These findings support the notion that, despite its benign classification, endometriosis harbours molecular traits typically associated with malignancy.

In this context, endometriosis is a powerful model for studying the early molecular changes involved in cellular transformation. Its intermediate status between physiological tissue remodelling and pathological proliferation offers a unique advantage for identifying regulatory mechanisms that either restrain or promote malignancy.

This refined molecular profiling may serve as a solid foundation for targeted genetic studies and therapeutic explorations. Understanding which mutations contribute to disease maintenance versus those that may trigger malignant progression could pave the way for novel biomarkers and intervention strategies for both endometriosis and cancer.

In this overview, endometriosis is not only a challenging clinical entity but also a valuable biological platform, capable of informing both benign disease management and oncological research.

## Conflict of interest

The authors declare no conflict of interest.

## Author contributions

CM: Conceptualization, data curation, formal analysis, methodology, writing—original draft, writing—review & editing; MCV, RS and EDG: data curation, methodology, writing—original draft; CFB, EDM, AG, and TDBR: Data curation, formal analysis, writing—review & editing; NB, AP, VG, AG, GB, AR, VB: Data curation, formal analysis, resources, supervision, validation, writing—review & editing; LI: Conceptualization, data curation, formal analysis, supervision, validation, writing—original draft, writing—review & editing. All authors have read and agreed to the published version of the manuscript.
